# Measurement of Individual Doses of Radiation by Personal Dosimeter Is Important for the Return of Residents from Evacuation Order Areas after Nuclear Disaster

**DOI:** 10.1371/journal.pone.0121990

**Published:** 2015-03-25

**Authors:** Makiko Orita, Naomi Hayashida, Yasuyuki Taira, Yoshiko Fukushima, Juichi Ide, Yuuko Endo, Takashi Kudo, Shunichi Yamashita, Noboru Takamura

**Affiliations:** 1 Department of Global Health, Medicine and Welfare, Nagasaki University Graduate School of Biomedical Sciences, Nagasaki, Japan; 2 Department of Radioisotope Medicine, Nagasaki University Graduate School of Biomedical Sciences, Nagasaki, Japan; 3 Department of Disaster Medicine, Atomic Bomb Disease Institute, Nagasaki University Graduate School of Biomedical Sciences, Nagasaki, Japan; 4 Nagasaki Prefectural Office, Nagasaki, Japan; 5 Department of Nursing, Hirosaki University Graduate School of Health Sciences, Hirosaki, Japan; 6 Kawauchi Municipal Government, Fukushima, Japan; Georgetown University, UNITED STATES

## Abstract

To confirm the availability of individual dose evaluation for the return of residents after the accident at the Fukushima Dai-ichi Nuclear Power Plant (FNPP), we evaluated individual doses of radiation as measured by personal dosimeters in residents who temporarily stayed in Evacuation Order Areas in Kawauchi village, which is partially located within a 20 km radius of the FNPP. We also compared individual doses with the external radiation doses estimated from the ambient dose rates and with doses estimated from the concentrations of radionuclides in the soil around each individual’s house. Individual doses were significantly correlated with the ambient doses in front of the entrances to the houses (r = 0.90, p<0.01), in the backyards (r = 0.41, p<0.01) and in the nearby fields (r = 0.80, p<0.01). The maximum cumulative ambient doses in the backyards and fields around the houses were 6.38 and 9.27 mSv/y, respectively. The maximum cumulative individual dose was 3.28 mSv/y, and the median and minimum doses were 1.35 and 0.71 mSv/y. The estimated external effective doses from concentrations of artificial radionuclides in soil samples ranged from 0.03 to 23.42 mSv/y. The individual doses were moderately correlated with external effective doses in the backyards (r = 0.38, p<0.01) and in the fields (r = 0.36, p<0.01); however, the individual doses were not significantly correlated with the external effective doses in front of the entrances (r = 0.01, p = 0.92). Our study confirmed that individual doses are low levels even in the evacuation order area in Kawauchi village, and external effective dose levels are certainly decreasing due to the decay of artificial radionuclides and the decontamination of contaminated soil. Long-term follow-up of individual doses as well as internal-exposure doses, environmental monitoring and reconstruction of infrastructure are needed so that residents may return to their hometowns after a nuclear disaster.

## Introduction

Due to the accident at the Fukushima Dai-ichi Nuclear Power Plant (FNPP) following the Great East Japan Earthquake on March 11, 2011, a large amount of radionuclides were released into the atmosphere. The estimated total release assumed by the United Nations Scientific Committee on the Effects of Atomic Radiation (UNSCEAR) for iodine-131 (^131^I), cesium-134 (^134^Cs) and cesium-137 (^137^Cs) were 120, 9.0 and 8.8 peta-becquerels (PBq), respectively. [1] In response to the accident, the Japanese government created an Evacuation Order Area for residents living within a 20 km radius of the FNPP. Furthermore, certain areas beyond the 20 km radius, where there remained concerns that cumulative doses of radiation might reach 20 mSv/y, were designated as Deliberate Evacuation Areas. In total, approximately 154,000 people have evacuated from their hometowns, among them 109,000 from the evacuation order area. [1–4]

**Fig 1:**
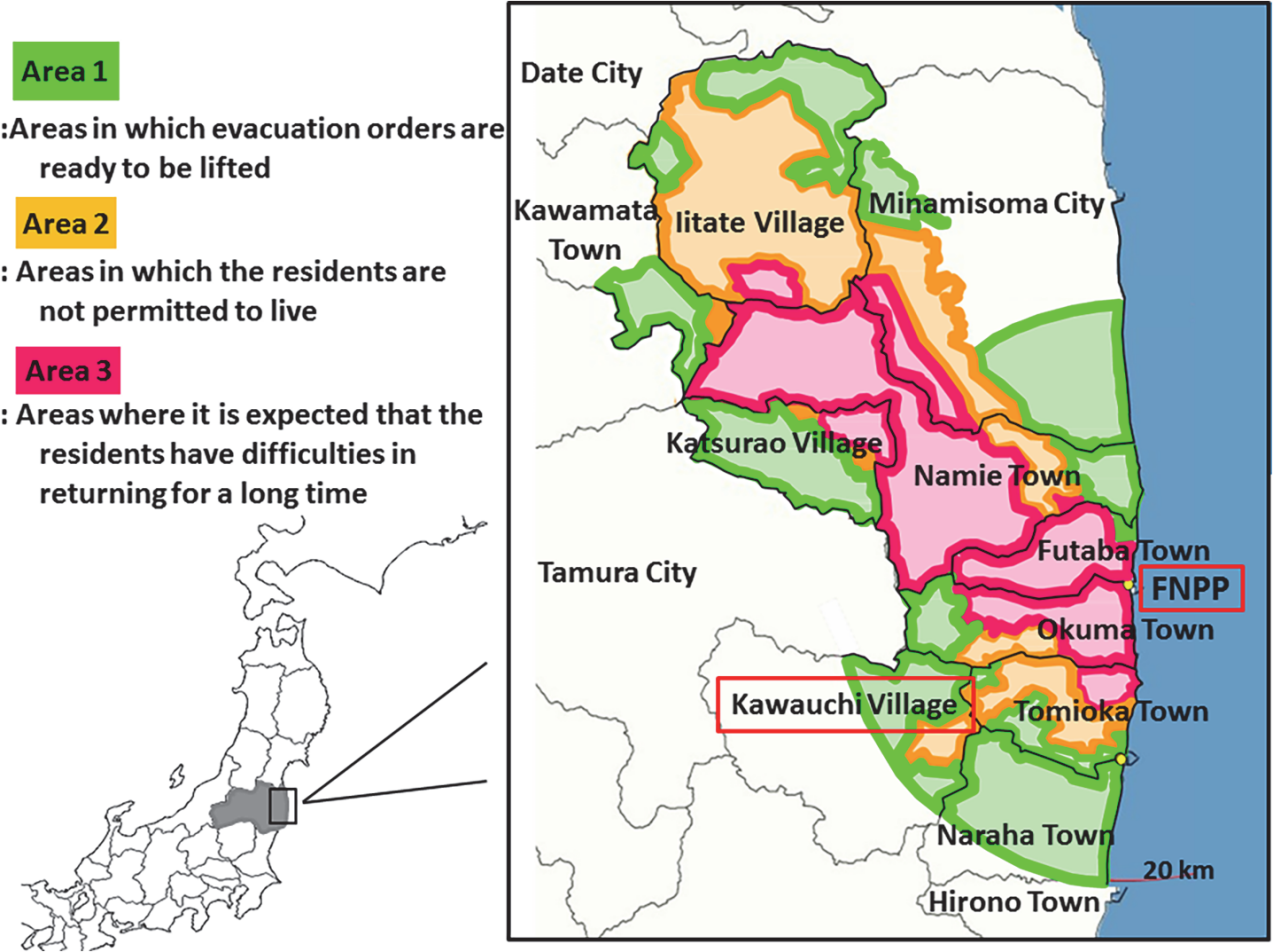
The Status of evacuation order areas in Fukushima Prefecture.

Both the Evacuation Order Area and the Deliberate Evacuation Area have been rearranged by the Japanese government, depending on the annual cumulative dose levels, and were reclassified into the following three areas on April 1, 2013: Area 1, where the evacuation orders are ready to be lifted; Area 2, where the residents are not permitted to live; and Area 3, where it is expected that the residents will have difficulties in returning for a long time (**Fig. 1**). [5]

These evacuation order areas were designated based on the exposure dose estimated from the ambient dose rates. [6, 7] However, it is known that the exposure doses estimated from the ambient doses might cause a significant “gap” with individual doses measured by personal dosimeter, because of factors such as shielding effects. In the initial phase of the accident at the FNPP, we evaluated the relationship between the reported ambient dose equivalent and the individual dose rate as recorded by medical staff in Fukushima City (within a 60 km radius of the FNPP), and showed that the individual dose is much lower than that determined ambient dose. [8] These results suggest that when we evaluate the radiation exposure dose of residents who will return to their hometowns located in the evacuation order area, it is important to measure individual dose by personal dosimeter. However, individual doses have not been estimated because residents are allowed only limited entry into these evacuation order areas.

In this study, we evaluated individual doses as measured by personal dosimeters in residents who temporarily stayed in evacuation order areas (Area 1 and Area 2) in the Kawauchi village, which is partially located within 20 km radius from the FNPP. We also compared individual doses with the external radiation doses estimated from the ambient dose rates and with doses estimated from the concentrations of radionuclides the soil around the residential houses to confirm the availability of individual dose evaluation for the return of residents after the nuclear disaster.

## Methods

### Ethics Statement

The study was approved by the ethics committee of Nagasaki University (project registration number 14011079), and written informed consent was obtained from all participants. Before the study, we obtained the permission from Kawauchi Municipal Government to measure ambient doses and collect soil samples in the village.

### Study Participants and Individual Dose Evaluation

The study was conducted in Kawauchi village, including areas in which evacuation orders are ready to be lifted (Area 1 of **Fig. 1**) and areas in which the residents are still not permitted to live (Area 2 of **Fig. 1**). Area 1 contained 274 residents and Area 2 contained 54 residents before the accident at the FNPP. At present, all residents are allowed only limited entry into these areas. They are temporarily permitted to return to their houses during holidays such as the New Year event; 56 residents had returned to their houses in August and December 2013.

Only 19 residents (10 men and 9 women, 57–83 years old) were included in this study. A personal dosimeter (MYDOSE G2, PDM-501, Hitachi-Aloka Medical, Tokyo, Japan) capable of measuring total dose ranges of 0.01 μSv to 1 Sv was used to determine the individual external doses. The sensitivity source at the dosimeter was calibrated with a ^137^Cs photon source. The enrolled study participants agreed to wear personal dosimeters during returns to their houses (for several days) except for while bathing. Personal dose data were calculated for each day (μSv/d) and multiplied by 365 days to calculate the annual cumulative individual dose (mSv/y).

### Ambient Dose Rate Measurements

Ambient dose rates outside the houses of study participants were measured 1 m above the ground using a scintillation survey meter (PDR-201, Hitachi-Aloka Medical, Ltd., Tokyo, Japan). The measured spots were in front of the entrances of the houses, in the backyards and in nearby fields such as rice fields. The survey meter was calibrated with a standard gamma-ray field. The annual cumulative dose (mSv/y) was calculated in accordance with the reports of the Ministry of the Environment's plan, and used the following formula:

[Annual cumulative dose(mSv/y)]=(Ambient dose rate)×(8h+16h×0.4)×(365/1000)

in which 8 h is the number of hours one stays outdoors per day, and 16 h is the number of hours one stays indoors per day. The result is multiplied by the coefficient 0.4 to account for the shielding effect of the buildings [9].

### Measurement of Radionuclides in Soil Samples

**Fig 2:**
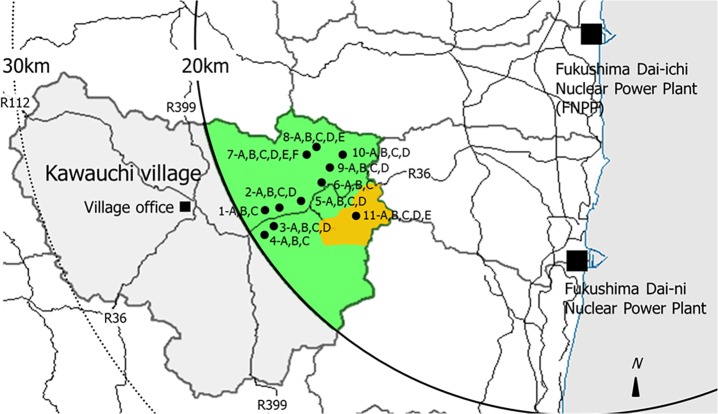
Location of sampling points, Kawauchi Village, Fukushima Prefecture. Closed circles (●) are sampling points.

In addition, 45 samples of surface soil after decontamination (0–5 cm) were collected at the same place where the ambient dose rates were measured. Places with collected samples are shown in **Fig. 2**. Soil sampling was carried out by a core sampling technique at all sampling locations. After collection, all samples were dried in a fixed temperature dryer (105°C, 24 hours). The samples were then sieved and the fine particles (< 2 mm) were prepared before measuring radionuclide activity.

After preparation, the soil samples were put into plastic containers and analyzed with a high purity germanium detector (ORTEC, GEM35, Ortec International Inc., Oak Ridge, TN, USA) coupled to a multi-channel analyzer (MCA7600, Seiko EG&G Co., Ltd., Chiba, Japan) for 3,600 s. The measuring time was set to detect the objective radionuclide, and the gamma-ray peaks used for the measurements were 604.66 keV for ^134^Cs (Half-life; 2.1 y), 661.64 keV for ^137^Cs (30 y) and 1460.75 keV for ^40^K (1.3 × 10^9^ y). Decay corrections were made based on the sampling data. Sample collection, processing and analysis were executed in accordance with the standard methods of radioactivity measurement authorized by the Ministry of Education, Culture, Sports, Science, and Technology in Japan (MEXT).

### Effective Dose

After the measurements, external effective doses were estimated with the following formula:

$$E_{ext}=\sum_{i=1}^{n}C_{g,i}\cdot {CF}_{4,i}$$

in which, C_*g*,*i*_ is the average deposition concentration of radionuclide *i* (radiocesium) [kBq/m^2^; estimated from the radiocesium concentration in Bq/kg-dry including soil particles (< 2 mm) and collected areas of surface soil]; CF_4,i_ is the conversion factor for exposure to ground contamination [(mSv/kBq/m^2^)]; and *n* is number of radionuclides (^134^Cs and ^137^Cs). To adjust the effective dose from deposition by taking into account shielding and partial occupancy, we used the following formula:

$$E_{est}^{po}=E_{est}\cdot \left[SF\cdot OF+\left(1-OF\right)\right]$$

in which SF is the shielding factor from measurements during occupancy [one and two story wood-frame house (no basement); 0.4]; and OF is the occupancy factor [outdoors for 8 hours, indoors for 16 hours a day]. Evaluation of the external effective dose was executed in accordance with the IAEA-TECDOC-1162. [10]

### Statistical Analysis

Analysis of variance was performed to compare accumulated individual doses with accumulated ambient doses. Simple linear regression analysis was performed to confirm the relationship between the personal dose and the external radiation dose. Because the personal dose and the external radiation dose were distributed in a skewed manner, logarithmic transformation was performed. Probability values less than 0.05 were considered indicative of statistical significance. All statistical analysis was performed using SPSS statistics 18.0 (SPSS Japan, Tokyo, Japan).

## Results

**Fig 3:**
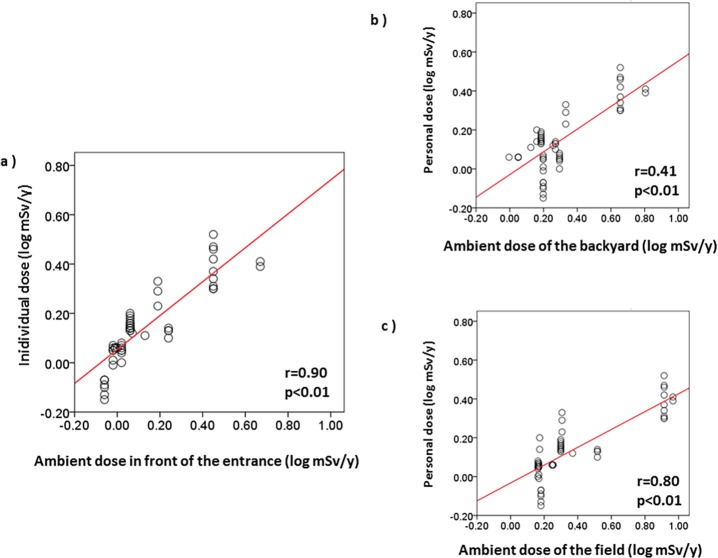
Relationship between individual doses and cumulative ambient doses.

Individual doses and the ambient doses in front of the entrances, in the backyards and in the fields are shown in **Table 1**. By simple linear regression analysis, individual doses were significantly correlated with the ambient doses in front of the entrances (r = 0.90, p<0.01), in the backyards (r = 0.41, p<0.01) and in the fields (r = 0.80, p<0.01) (**Fig. 3**). The regression equations used for the areas in front of the entrances, in the backyards and in the fields were calculated as follows: [the individual dose (mSv/y)] = 0.548×[the ambient dose (mSv/y)]+0.652, [the individual dose (mSv/y)] = 0.362×[the ambient dose (mSv/y)]+0.674, [the individual dose (mSv/y)] = 0.191×[the ambient dose (mSv/y)]+0.893, respectively.

**Table 1:** The ambient doses estimated from the ambient dose rates and individual doses using electric personal dosimeter.

	Median (minimum-maximum) (mSv/y)
Air doses in front of the entrance	1.16 (0.88–4.73)
Air doses in backyard	1.58 (0.99–6.38)
Air doses in field	1.99 (1.45–9.26)
Individual doses	1.35 (0.71–3.28)

**Fig 4:**
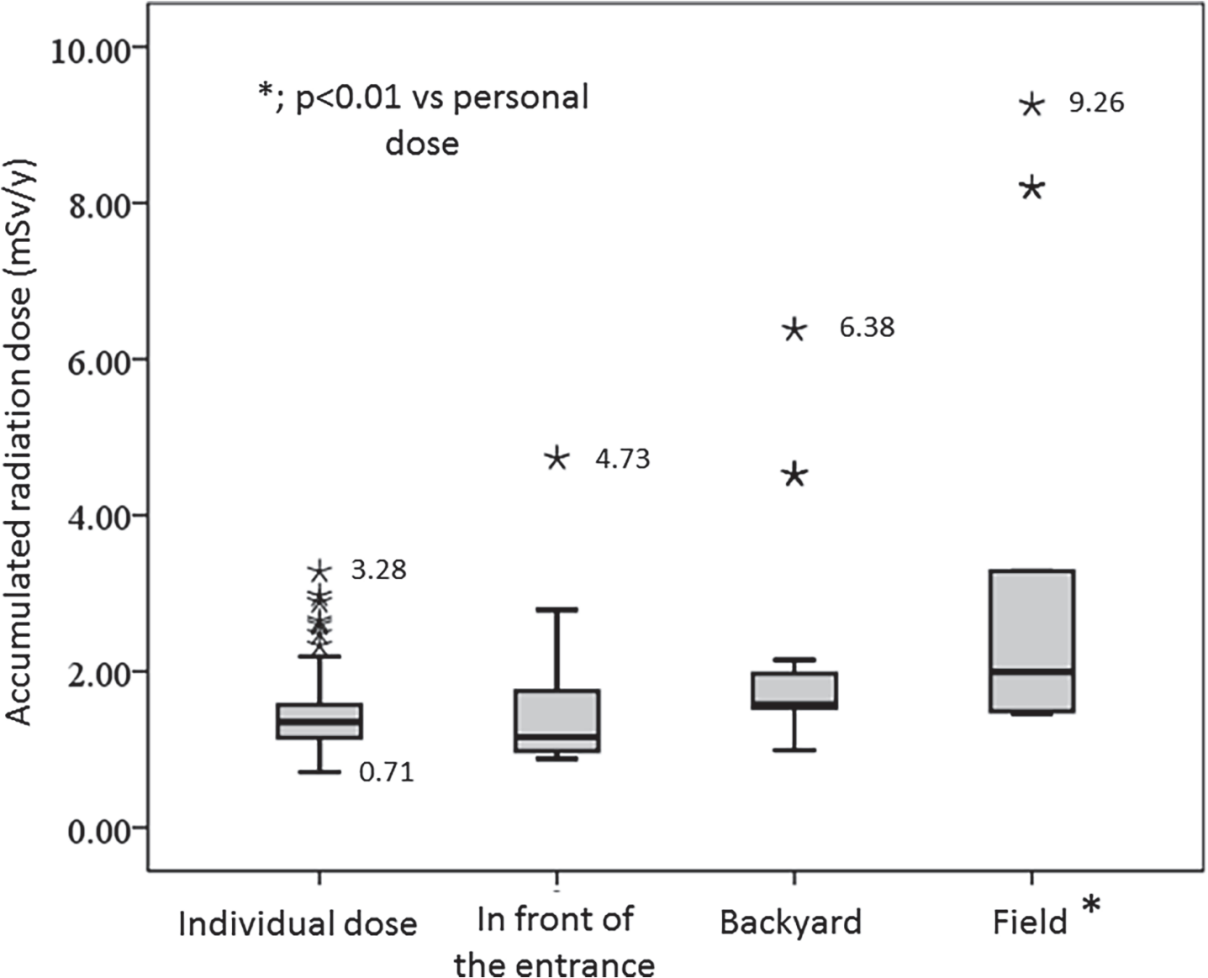
Cumulative individual doses, cumulative ambient doses in front of the entrance, in the backyard, and field around the houses.

The cumulative individual doses, as well as the cumulative ambient doses in front of the entrances, in the backyards and in the fields around the houses are shown in **Fig. 4**. The maximum cumulative ambient doses in the backyards and fields around the houses were 6.38 and 9.27 mSv/y, respectively. The maximum cumulative individual dose was 3.28 mSv/y, and the median and minimum doses were 1.35 and 0.71 mSv/y, respectively. The cumulative individual doses were significantly lower than the accumulated ambient doses in the fields (p<0.001).

The concentrations of artificial radionuclides in the soil, the estimated external effective doses from surface soils due to radiocesium and the ambient dose rates and radiocesium ratios (^134^Cs/^137^Cs) are shown in **Table 2**. The prevalent dose-forming artificial radionuclides from all samples were ^134^Cs and ^137^Cs. Concentrations of radiocesium exceeded 8,000 Bq/kg-dry (which is the standard value for storing decontamination waste by Japanese guidelines) in 5 soil samples (2-D, 3-B, 7-B, 10-D and 11-D). Radiocesium ratios (^134^Cs/^137^Cs) ranged from 0.30 to 0.52 at the time of sampling. The estimated external effective doses ranged from 0.03 to 23.42 mSv/y.

**Table 2:** Distribution of detected artificial radionuclides in soil samples.

No.	Spot	^134^Cs concentration (Bq/kg-dry)	^137^Cs concentration (Bq/kg-dry)	^40^K concentration (Bq/kg-dry)	External effective dose (mSv/y)	Ambient dose rate (μSv/h)	Ratio (^134^Cs/^137^Cs)
1-A	^a^E	384 ± 13.0^e^	794 ± 17.9	849 ± 73.0	0.85	0.18	0.48
1-B	^b^B	1,130 ± 26.4	2,410 ± 36.0	866 ± 86.7	2.54	0.30	0.47
1-C	^c^F	1,220 ± 20.6	2,470 ± 27.8	793 ± 64.3	2.69	0.28	0.49
2-A	E	10.4 ± 2.54	34.6 ± 3.36	879 ± 57.1	0.03	0.17	0.30
2-B	B	124 ± 7.60	278 ± 10.9	855 ± 73.8	0.29	0.30	0.45
2-C	F	371 ± 12.2	911 ± 18.9	835 ± 70.0	0.89	0.29	0.41
2-D	^d^R	3,530 ± 58.3	8,100 ± 85.3	1,010 ± 126	8.21	0.33	0.44
3-A	E	86.2 ± 6.10	205 ± 8.52	935 ± 70.2	0.20	0.19	0.42
3-B	B	6,810 ± 45.4	15,900 ± 68.6	704 ± 58.9	15.97	0.19	0.43
3-C	F	537 ± 14.8	1,170 ± 21.3	905 ± 75.0	1.22	0.34	0.46
3-D	R	421 ± 11.4	978 ± 17.0	985 ± 66.9	0.99	0.28	0.43
4-A	E	88.3 ± 4.97	199 ± 7.04	742 ± 51.8	0.20	0.26	0.44
4-B	B	139 ± 6.25	287 ± 8.50	923 ± 58.3	0.31	0.25	0.48
4-C	R	147 ± 6.73	282 ± 8.76	948 ± 61.0	0.32	0.29	0.52
5-A	E	272 ± 13.5	610 ± 19.4	740 ± 90.0	0.63	0.33	0.45
5-B	B	133 ± 8.79	362 ± 12.9	1,000 ± 83.5	0.34	0.36	0.37
5-C	F	1,130 ± 25.9	2,730 ± 39.4	800 ± 87.6	2.69	0.63	0.41
5-D	R	571 ± 18.5	1,310 ± 27.5	809 ± 85.2	1.33	0.3	0.44
6-A	E	296 ± 10.5	720 ± 16.1	802 ± 68.2	0.71	0.22	0.41
6-B	B	1,680 ± 30.3	3,790 ± 44.9	816 ± 85.9	3.88	0.29	0.44
6-C	F	223 ± 9.92	541 ± 15.1	698 ± 67.1	0.53	0.38	0.41
7-A	E	1,540 ± 25.5	3,550 ± 37.6	775 ± 71.4	3.59	0.2	0.43
7-B	B_1_	3,330 ± 36.7	7,810 ± 55.8	871 ± 75.4	7.83	0.38	0.43
7-C	F_1_	290 ± 11.3	598 ± 15.4	959 ± 76.1	0.64	0.28	0.48
7-D	R	396 ± 10.5	896 ± 15.7	826 ± 60.2	0.92	0.27	0.44
7-E	B_2_	2,020 ± 30.2	4,410 ± 42.7	870 ± 77.4	4.60	0.56	0.46
7-F	F_2_	399 ± 12.9	767 ± 17.1	837 ± 70.7	0.86	0.39	0.52
8-A	E	98.9 ± 5.32	199 ± 7.28	789 ± 55.5	0.22	0.19	0.50
8-B	B	387 ± 13.5	958 ± 20.5	1,020 ± 83.3	0.93	0.21	0.40
8-C	F_1_	365 ± 12.6	821 ± 18.4	846 ± 75.4	0.84	0.31	0.44
8-D	F_2_	233 ± 9.47	499 ± 13.3	1,070 ± 77.6	0.53	0.34	0.47
8-E	F_3_	880 ± 17.7	2,060 ± 26.8	849 ± 67.4	2.07	0.34	0.43
9-A	E	1,280 ± 25.3	2,970 ± 37.9	679 ± 73.3	2.99	0.22	0.43
9-B	B	414 ± 13.4	1,030 ± 20.6	855 ± 74.3	1.00	0.28	0.40
9-C	R	968 ± 18.4	2,240 ± 27.6	955 ± 71.6	2.26	0.66	0.43
9-D	F	845 ± 17.6	1,960 ± 26.6	858 ± 71.2	1.98	0.28	0.43
10-A	E	654 ± 17.2	1,610 ± 26.3	865 ± 76.6	1.57	0.29	0.41
10-B	B	1,010 ± 42.2	2,500 ± 34.0	688 ± 75.5	2.43	0.41	0.40
10-C	F	962 ± 23.2	2,180 ± 33.9	700 ± 78.5	2.23	0.39	0.44
10-D	R	10,100 ± 83.3	23,000 ± 124	693 ± 99.4	23.42	0.82	0.44
11-A	E	271 ± 9.45	587 ± 13.5	474 ± 48.0	0.61	0.53	0.46
11-B	B	2,120 ± 29.8	5,010 ± 45.2	623 ± 65.5	5.00	0.86	0.42
11-C	F_1_	2,310 ± 26.5	5,460 ± 40.2	696 ± 59.4	5.45	1.56	0.42
11-D	F_2_	2,560 ± 28.5	6,080 ± 43.1	884 ± 67.0	6.05	1.56	0.42
11-E	R	2,340 ± 29.4	5,590 ± 44.5	902 ± 70.9	5.55	0.86	0.42

Abbreviation:

^a^E = Front of the entrance of house,

^b^B = The backyard of house,

^c^F = The field like a rice field,

^d^R = The road near side of the house.

^e^; Error shows one sigma standard deviation from counting statistics.

**Fig 5:**
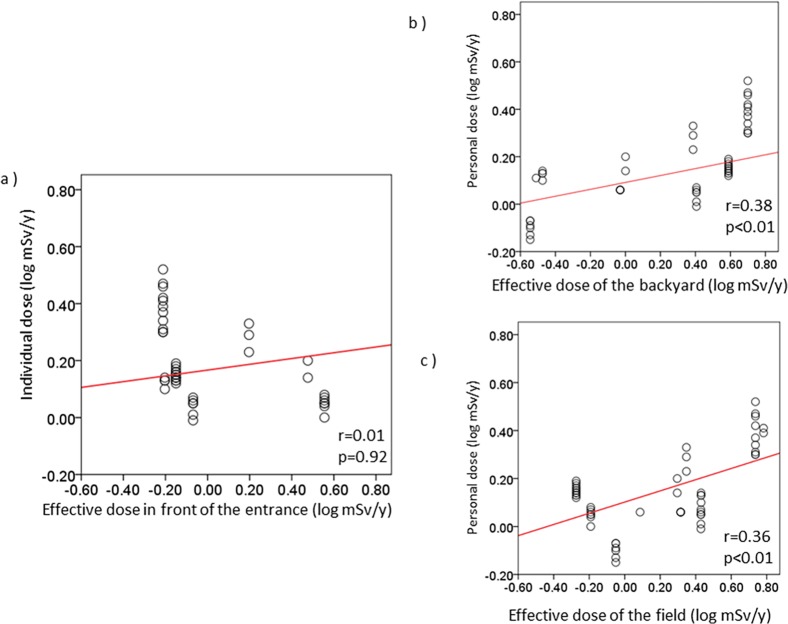
Relationship between individual doses and effective doses from soil samples.

By simple linear regression analysis, individual doses were moderately correlated with external effective doses in the backyards (r = 0.38, p<0.01) and in the fields (r = 0.36, p<0.01) (**Fig. 5**). However, individual doses were not significantly correlated with external effective doses in front of the entrances (r = 0.01, p = 0.92).

## Discussion

Worldwide experiences following nuclear and non-nuclear accidents have shown that both nations and individuals are not willing to leave affected areas. [11] Kawauchi village is mainly located 20–30 km southwest of the FNPP and was partially included in the Evacuation Order Area (within a 20 km radius of the FNPP). Almost all residents were evacuated from the village at the initial phase of the accident at the FNPP. On January 31, 2012, the head of Kawauchi village declared that residents who resided at least 20 km away from the FNPP could return to their homes because the Japanese Prime Minister had declared that the FNPP reactors had achieved a state of “cold shutdown” in December 2011. [12] After the declaration, the village office has been working steadily toward reconstruction, including decontamination within the village, the reopening of elementary and junior high schools and of commercial facilities and the restarting of agriculture. On the other hand, many residents of the village remained concerned about radiation exposure and health effects. [12] As of June 1, 2014, only 1,278 of 2,746 (46.5%) residents had returned to the village, and residents who resided within a 20 km radius of the FNPP were not permitted to return. According to the results of the current study, Kawauchi village has lifted the evacuation order for Area 1 and allowed residents who resided there to return beginning in October 2014.

In November 2013, the Nuclear Regulation Authorities of the Japanese government suggested that new radiation management measures should be implemented on the basis of individual dose measurements. [13] We believe that the current case of Kawauchi village would be a model for this suggestion.

This is the first report to evaluate individual doses measured by personal dosimeters in residents within a 20 km radius of the FNPP, and we showed that the cumulative individual doses are lower than those estimated from the ambient doses. Radiation exposure to a person is the result of exposure to the radiations in the environment. It is estimated that the discrepancy between cumulative individual doses and those estimated from ambient doses is due mainly to variations in the daily lives of residents, differing shield-rates of houses and characteristics of the dosimeters, such as directional dependency of the irradiation angle.

We also showed that the cumulative individual doses were significantly correlated with the ambient doses in front of the entrances, in the backyards, and in the fields. Individual doses were especially strongly correlated with the ambient doses in front of the entrances. On the other hand, individual doses were moderately correlated with external effective doses calculated from soil concentrations of in the backyards and in the fields, although there was no correlation between the individual doses and the external effective doses in front of the entrances, probably due to the effects of decontamination of soils around residential houses. In Kawauchi village, decontamination of residential houses has been conducted since mid-2011, even within the 20 km radius of the FNPP. Large land remediation programs involving decontamination of residential areas have also been implemented in affected areas of Fukushima prefecture. The United Nations Scientific Committee on the Effects of Atomic Radiation (UNSCEAR) reported that these programs may have the potential to reduce future exposure of the public residing in the more affected areas and detailed information is needed to assess the efficiency of the land remediation actions [1]. A decontamination policy should take priority in areas where decontamination is required from the view point of human health; thus, decontamination work was conducted in accordance with the technical guidelines for decontamination developed to complement the ordinance of the Ministry of the Environment of Japan. [14] Namely, within a 20 m radius from the residential houses, forest lands have been cleared of branches, fallen leaves, and leaf mold. The surface soil of the backyards and fields has been removed to a 5 cm depth, and roofs and walls of residential houses have been washed with high pressure cleaning equipment. The surface soil in front of the entrances has been removed to a 5 cm depth and the soil has been top dressed. These steps have reduced the concentrations of radionuclides in the surface soil around the entrances. Our current results suggest that the exposure doses estimated from the air dose rates around the residents’ houses were mainly derived from areas where some radioactive substances remained after the decontamination and from areas that were outside the sites where decontamination had been implemented.

Since the accident, measurements of external and internal exposure doses of residents in Fukushima Prefecture outside a 20 km radius of FNPP have been reported [15–21]. The Fukushima Health Survey conducted by the Fukushima Prefecture estimated the external radiation doses of residents based on their behavior during the four months following the accident (March 11, 2011 to July 11, 2011). [15, 16] In this survey, 1,379 residents who resided in Kawauchi village at the time of the accident were also analyzed. The analysis revealed that 1,009 (73.1%) were exposed <1 mSv, 1,358 (98.5%) were exposed <2 mSv and 1,375 (99.7%) were exposed <3 mSv. [16] In August and September 2012, Harada et al. evaluated the personal external dose equivalent of 382 residents who lived 20 km away from the FNPP in the village using Quixel Badge (Nagase Landauer, LTD, Tsukuba, Japan), and showed that the residents’ cumulative doses ranged from 0.04 to 1.2 mSv/2 months, with a median of 0.15 mSv/2 months. [17] In addition, Tsubokura et al. reported levels of internal cesium exposure among the Kawauchi villagers using a whole body counter from April 2012 to March 2013 and reported that their levels of internal radiation exposure remained very low even in the highly radiation-contaminated region at the time of the nuclear disaster. [18] These findings suggest that external and internal doses from the accident at the FNPP were relatively limited in the general population, and our current results showed that external doses within the 20km zone of Kawauchi village are also limited.

There are several limitations in this study. First, the study was conducted with a relatively small sample. The total population residing within 20km of the FNPP in Kawauchi village was only 300 even before the accident. Among these, 56 residents returned to their homes during the study period; however, we were only able to include 19 residents in this study because the other 37 residents spent too few days at their homes to allow assessment of their individual doses. While the study population size was relatively small, we believe that our study has merit as it confirms the importance of the measurement of individual doses for supporting the residents who desire to return to their homes that are located in the evacuation order area (i.e., within a 20 km radius of the FNPP). Second, we evaluated only soil samples and measured ambient dose rates only outside the house to determine the effectiveness of the decontamination; evaluation of environmental doses at the entrances, backyards, fields, and in particular, inside the houses might also be needed. Third, the number of collected soil samples was limited. Since radionuclides in soil samples may be unequally distributed around the FNPP, continuous sample collection is definitely needed.

In conclusion, we confirmed the availability of individual-dose evaluation for returning residents after a nuclear disaster. Although the safety of a radiation-contaminated area should be determined initially based on radiation doses estimated from ambient dose rates, measurement of individual doses also is an effective way to support residents who desire to return to homes located in the evacuation order area. Long-term follow-up of individual doses as well as internal-exposure doses in consideration of metabolic factors, environmental monitoring and reconstruction of infrastructure are needed so that residents may return to their hometowns after a nuclear disaster. Finally, it is important to implement risk communications with residents, based on the data of actually measured individual doses to address the residents’ anxieties about radiation exposure. The case of Kawauchi village will be the initiative model for the return of residents to their homes and for the reconstruction after the FNPP accident.
